# Efficacy of ultra-early rehabilitation on elbow function after Slongo’s external fixation for supracondylar humeral fractures in older children and adolescents

**DOI:** 10.1186/s13018-021-02671-4

**Published:** 2021-08-21

**Authors:** Man He, Qian Wang, Jingxin Zhao, Yu Wang

**Affiliations:** 1 Department of Rehabilitation, Affiliated Hospital of Chengde Medical College, Chengde, Hebei 067000 People’s Republic of China; 2 Trauma Department of Orthopedics, Affiliated Hospital of Chengde Medical College, 36 Nanyingzi Street, Shuangqiao District, Chengde, Hebei 067000 People’s Republic of China

**Keywords:** Supracondylar humeral fracture, Older children, Adolescent, Rehabilitation, External fixation

## Abstract

**Objective:**

To evaluate the efficacy of ultra-early rehabilitation on elbow function after Slongo’s external fixation for supracondylar humeral fractures in older children and adolescents.

**Methods:**

We retrospectively analyzed clinical data from 49 older children (> 8 years) and adolescents with supracondylar humerus fracture who were treated with Slongo’s external fixation in our hospital from January 2016 to August 2020. Twenty-three patients received ultra-early postoperative rehabilitation (rehabilitation group) and 26 patients were not subjected to postoperative rehabilitation (control group). Time to ROM required for functional activity of daily living(ROM-ADL) in both groups was recorded postoperatively. Patients were followed up at 3 and 6 months after surgery to compare the elbow range of motion (ROM) and carrying angle (CA). Postoperative complications were assessed in both groups. Flynn scores and modified Mayo Elbow Performance Scores were also performed.

**Results:**

The elbow function at 6 months after surgery was significantly better than that at 3 months in the control group (*P* = 0.32). Time to ROM-ADL in the rehabilitation group was significantly shorter than that of the control group (*P* = 0.028). The elbow function, Flynn scores, and modified Mayo scores in the rehabilitation group at 3 and 6 months after surgery were significantly better than that of the control group (*P*(ROM, 3 months and 6 months) = 0.012 vs 0.039; *P*(Flynn scores, ROM, 3 months and 6 months) = 0.028 vs 0.005; *P*(Flynn scores, CA, 3 months and 6 months) = 0.032 vs 0.026; *P*(Modified Mayo scores, 3 months and 6 months) = 0.039 vs 0.024; respectively). There were no iatrogenic injuries, secondary fracture displacements, myositis ossificans, elbow deformities, or other complications in either group. One case of nail tract infection occurred in the rehabilitation group and was cured.

**Conclusion:**

Slongo’s external fixation is a safe and effective surgical treatment for supracondylar humeral fractures in children over 8 years old and adolescents. Ultra-early rehabilitation treatment for postoperative children can significantly speed up the recovery after surgery.

## Introduction

Supracondylar humeral fracture is the most common elbow fracture in children. It usually occurs in children between the ages of 5 and 7 [1, 2]. Supracondylar humeral fractures can be divided into extension and flexion fractures and approximately 97% are extension type fractures [3]. Supracondylar humeral fractures are commonly divided into three types according to the modified Gartland classification, while surgical treatment is recommended for Gartland II and III fractures in children [4]. The incidence of supracondylar humeral fractures in children has increased by 28% in the past decade [5], while the surgical rate has also increased [6]. There are several surgical techniques including K-wire, corticoid screw, and elastic intramedullary nail [7, 8]; however, the standard surgical treatment for displaced pediatric supracondylar humeral fractures is closed reduction and percutaneous pinning (CRPP), with the placement of a postoperative plaster cast [9, 10]. The elbow joint movement can be restricted to varying degrees after the removal of the plaster cast. Interestingly, children tend to not move the affected limb due to fear or pain. Therefore, the occurrence of elbow joint stiffness after surgery has become a concern of parents. For older children and adolescents, the recovery period is prolonged, and the risk of joint stiffness increases [11]. As a relatively new surgical method, external fixation has become a treatment choice for supracondylar humeral fracture in older children and adolescents. Indeed, this technique can enable children and adolescents to perform early elbow joint functional exercises after surgery, thus reducing the risk of elbow stiffness.

Therefore, the purpose of this study was to assess the effects of Slongo’s external fixation in the treatment of supracondylar humeral fractures in older children and adolescents. Moreover, we sought to assess the clinical value of ultra-early rehabilitation after external fixation operation in older children and adolescents.

## Methods

### Patients

We retrospectively analyzed clinical data of 49 older children (> 8 years) and adolescents with supracondylar humeral fractures who were treated with closed reduction and Slongo’s external fixation in our hospital from January 2016 to August 2020. A total of 23 patients were subjected to ultra-early postoperative rehabilitation (rehabilitation group), while 26 patients without postoperative rehabilitation were served as the control group. Children with open fractures, coexisting fractures of other parts, and injuries of other organs were excluded. Health education on disease, operation, and rehabilitation was carried out for children and their parents during the perioperative period. The informed consent of the parents was obtained prior to inclusion in the study.

### Surgical procedures

**Fig. 1:**
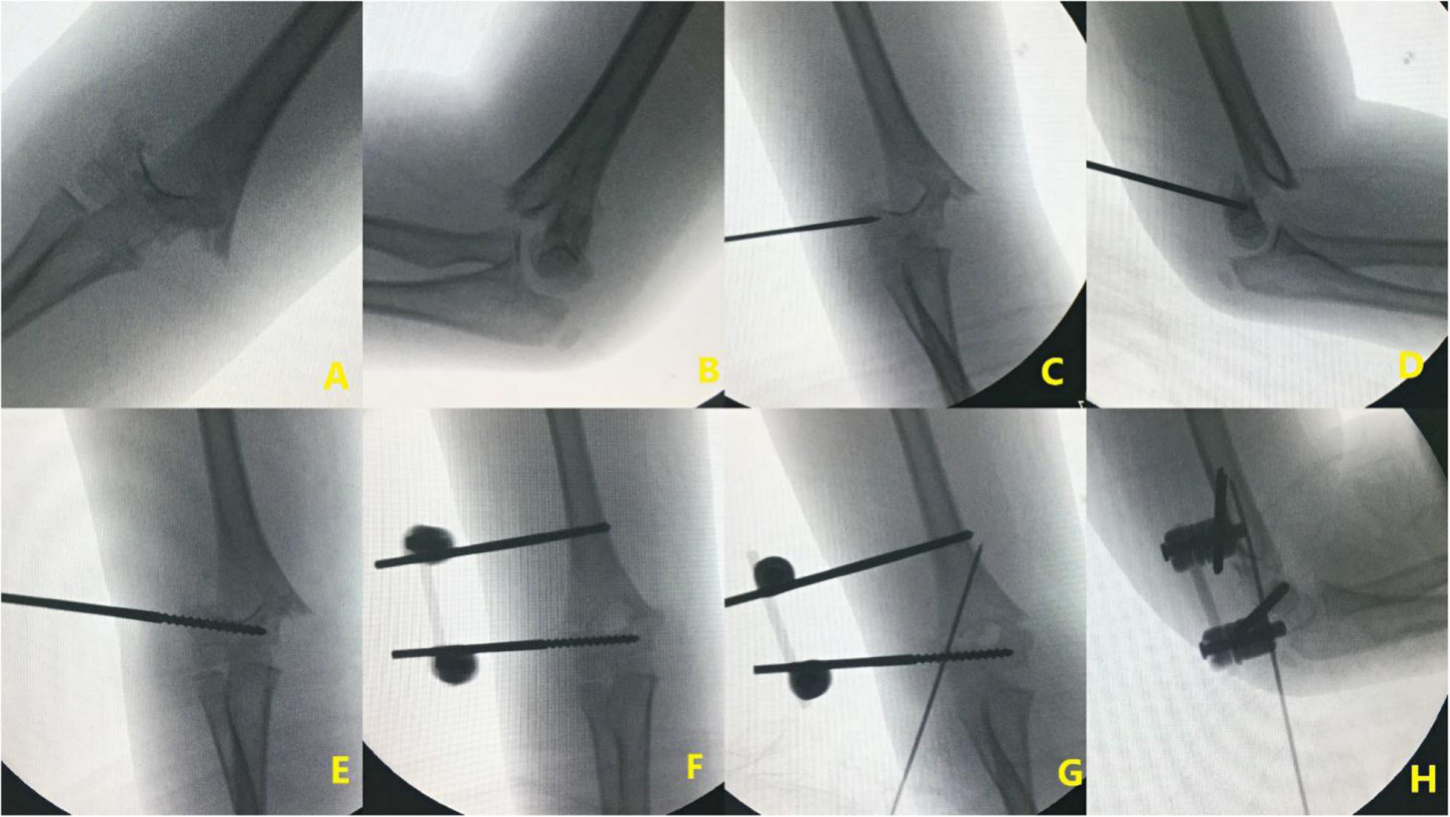
Preoperative X-ray radiographs (**A**, **B**); location and placement of the distal Schanz screw (**C**–**E**); placement of proximal Schanz screw (**F**); anteroposterior and lateral X-ray radiographs after anti-rotating Kirschner wire placement (**G**, **H**)

The operation was performed under general anesthesia. The patient was in the supine position and the fracture reduction was performed with a C-arm fluoroscopy. Distal 4.0-mm Schanz screw was used and was located above the external humeral condyle epiphyseal plate and in the rotation center of the elbow joint. The surgeon was careful to avoid screw injury to the epiphyseal and epiphyseal plate of the lateral condyle and to prevent the screw from entering the coronal fossa, olecranon fossa, and breaking through the cortex of the distal medial humerus. A proximal Schanz screw with a diameter of 4.0 mm was directly drilled into the humerus after a small incision. A sleeve was used to protect the surrounding soft tissue while drilling, and the screw was positioned at the center of the humerus. After the closed reduction was made, a connecting rod was used to fix the screws. Finally, a 1.8-mm or 2.0-mm Kirschner wire was inserted in the lateral condyle of the humerus to prevent rotation of the fracture end, with care to not limit elbow movement. Postoperative plaster casts were not used (Fig. 1).

### Postoperative rehabilitation

#### Rehabilitation group

The rehabilitation team of our hospital (including rehabilitation doctors, physical therapists, and occupational therapists) performed a postoperative systematic rehabilitation treatment. Active and passive activity training of adjacent joints on the day after surgery were performed, with active flexion and extension exercises of the affected shoulder, wrist, metacarpophalangeal joint, and interphalangeal joint. Elbow joint loosening + progressive resistance training (30 min/day, starting on the second day after surgery) was performed through passive flexion and extension of the elbow on the affected side. The elbow resistance training with an elastic band was performed starting 3 days after surgery on the premise that the patient had no significant pain. Wax therapy (15 min/day, performed before elbow joint loosening) was also conducted, using an appropriate wax size wax to wrap the elbow above 1 cm away from the Schanz screws. Occupational therapy (30 min ×2/day) was started 3 days after surgery and the child’s upper limbs were treated with fun occupational therapy using a RAMGUIDER upper limb rehabilitation training system, in which a suitable upper limb movement goal for the patient’s joint range of motion was set and, if painless, the overall active movement ability of the affected upper limb progressively increased through purpose-oriented training. The movement mode was adjusted according to the patient’s measured elbow joint range of motion every day. Lymphatic drainage of the affected limb (15 min/day) was performed by the physiotherapist on the affected upper limb of the patient after joint loosening and active resistance training every day. Finally, cold therapy (15 min/day) was performed after the abovementioned treatments were finished. Attention was paid to avoid secondary fracture displacements and the occurrence of myositis ossificans. The rehabilitation treatment lasted for 2 weeks, 6 days per week. After the patient and their parents had mastered the exercise methods, they were instructed to conduct exercises at home.

The control group did not receive ultra-early rehabilitation treatment after surgery. Patients that met the discharge criteria were discharged from the hospital and underwent self-rehabilitation training at home.

When discharged from the hospital, patients and parents in both groups were required to add a review WeChat public account to communicate with surgeons and rehabilitation doctors about their rehabilitation and exercises at any time through the form of WeChat video, so that they could receive timely guidance. Educational booklets for exercise in postoperative rehabilitation were given to the patients in both groups. Regular outpatient follow-up was conducted and suggestions on postoperative rehabilitation were given. The external fixation was removed after fracture healing.

### Follow-up indicators

Follow-up clinical examination and X-rays were taken at 3 and 6 months after the surgery. Range of motion (ROM), time to ROM required for functional activity of daily living (ROM-ADL), and carrying angle (CA) of the elbow were recorded. ROM-ADL was defined as 30°of extension, 130°of flexion [12]. Postoperative complications such as infection, iatrogenic nerve injury, ischemic contracture, myositis ossificans, and elbow varus/valgus deformities were monitored. Flynn scores and modified Mayo elbow function scores were performed for both groups.

### Statistical methods

The SPSS22.0 software was used to process the data. Measurement data were expressed as *x*^*2*^ ± *S*. Paired *t-*tests were used for comparisons of normally distributed data, while independent sample *t-*test was used for comparison between groups. Non-parametric chi-square was used to compare data that did not conform to a normal distribution. *P* < 0.05 was considered statistically significant.

## Results

There was no significant difference in basic information such as age, sex, height, and weight between the two groups (*P* > 0.05), as shown in Table 1. The elbow function of children in the control group showed a gradual improvement at 3 and 6 months (*P* < 0.05, Table 2). Time to ROM-ADL in the rehabilitation group was significantly shorter than that of the control group (*P* < 0.05, Table 1). The elbow function, Flynn scores, and modified Mayo scores of the elbow in the rehabilitation group at 3 and 6 months after surgery were significantly better than those of the control group (*P* < 0.05, Tables 2, 3, and 4). One case of nail tract infection occurred in the rehabilitation group, which was a superficial pin tract infection around the proximal Schanz screw. After local dressing changes for 1 week, the patient recovered. We detected no cases of pin tract infection with the anti-rotation wire. No cases of deep infection or osteomyelitis occurred. All patients with preoperative nerve injury recovered spontaneously after surgery. There were no other iatrogenic injuries, myositis ossificans, secondary fracture displacement, elbow deformities, or other complications in either group.

**Table 1:** Basic information of two groups

Group	The control group	Rehabilitation group	*P*
Gender(male/female)^a^	16:10	11:12	0.564
Age (years)	10.58 ± 1.68	11.14 ± 1.52	0.892
Height (cm)	121.33 ± 21.991	108.39 ± 23.259	0.860
Weight (kg)	32.95 ± 16.96	35.63 ± 12.68	0.140
Affected side (left / right)	9:17	8:15	0.470
Type^a^			0.284
Gartland II	10(38.46%)	6(26.09%)	
Gartland III	16(61.54%)	17(73.91%)	
Time from admission to operation (h)	35.81 ± 27.958	59.69 ± 38.969	0.151
Hospitalization time (days)	3.81 ± 0.168	4.78 ± 0.257	0.807
Operation time (min)	49.09 ± 4.153	50.62 ± 3.375	0.625
Number of intraoperative fluoroscopy (time)	23.81 ± 2.827	22.97 ± 1.858	0.416
Time to ROM-ADL (week)	6.65 ± 0.80	9.74 ± 1.25	0.028

^a^Chi-square test

**Table 2:** Comparison between the control group and the rehabilitation group after external fixator operation

	The control group		Rehabilitation group		*P*_*1*_	*P*_*2*_	*P*_*3*_
*n*	26		23				
	3 months after surgery	6 months after surgery	3 months after surgery	6 months after surgery			
Flexion in affected side (°)	139.88 ± 4.99	141.62 ± 6.81	140.76 ± 3.13	143.71 ± 3.26	0.017	0.007	0.018
Contralateral flexion (°)	146.25 ± 3.64	146.25 ± 3.64	146.23 ± 4.25	146.26 ± 3.25	0.89	0.92	1.46
Extension in affected side (°)	2.51 ± 1.09	2.04 ± 0.76	-1.23 ± 0.69	-2.29 ± 0.29	0.028	0.02	0.026
Contralateral extension (°)	-2.56 ± 1.01	-2.56 ± 1.01	-2.66 ± 2.45	-2.76 ± 2.25	0.76	0.83	1.23
Total ROM (°)	137.08 ± 1.72	138.38 ± 1.13	140.09 ± 2.61	141.74 ± 1.74	0.012	0.039	0.032
CA(°)	4.89 ± 0.82	8.13 ± 0.82	6.52 ± 1.66	9.94 ± 0.83	0.038	0.032	0.022

*P*_*1*_, comparison of *P* value between the two groups 3 months after surgery; *P*_2_, *P* value of the control group and the rehabilitation group 6 months after surgery; *P*_3_, comparison of *P* values between the rehabilitation group 3 months after surgery and the rehabilitation group 6 months after surgery

**Table 3:** Comparison of Flynn scores in two groups

Flynn scores	The control group		Rehabilitation group		*P*_1_	*P*_2_	*P*_3_
	3 months after surgery	6 months after surgery	3 months after surgery	6 months after surgery			
According to CA^a^					0.032	0.026	0.04
Excellent	1 (3.8%)	4 (15.4%)	9 (39.1%)	13 (56.5%)			
Good	24 (92.3%)	21 (80.8%)	13 (56.5%)	9 (39.1%)			
Fair	1 (3.8%)	1 (3.8%)	1 (4.3%)	1 (4.3%)			
Poor	0	0	0	0			
According to loss of ROM ^a^					0.028	0.005	0.029
Excellent	2 (7.7%)	6 (23.1%)	7 (30.4%)	12 (52.2%)			
Good	23 (88.5%)	19 (73.1%)	15 (65.2%)	10 (43.5%)			
Fair	1 (3.8%)	1 (3.8%)	1 (4.3%)	1 (4.3%)			
Poor	0	0	0	0			

*P*_*1*_, comparison of *P* value between two groups 3 months after surgery; *P*_2_, *P* value of the control group and the rehabilitation group 6 months after surgery; *P*_3_, comparison of *P* values between the rehabilitation group 3 months after surgery and the rehabilitation group 6 months after surgery

^a^Chi-square test

**Table 4:** Comparison of postoperative modified Mayo scores in two groups

Modified Mayo scores^a^	The control group		Rehabilitation group		*P*_1_	*P*_2_	*P*_3_
	3 months after surgery	6 months after surgery	3 months after surgery	6 months after surgery	0.039	0.024	0.043
Excellent	2 (7.7%)	6 (23.1%)	7 (30.4%)	13 (56.52%)			
Good	23 (88.5%)	19 (73.1%)	15 (65.2%)	9 (39.13%)			
Fair	1 (3.8%)	1 (3.8%)	1 (4.3%)	1 (4.3%)			
Poor	0	0	0	0			

^a^Chi-square test

*P*_1_, comparison of *P* value between two groups 3 months after surgery; *P*_2_, *P* value of the control group and the rehabilitation group 6 months after surgery; *P*_3_: Comparison of P values between the rehabilitation group 3 months after surgery and the rehabilitation group 6 months after surgery

## Discussion

### Application of external fixation in the treatment of supracondylar humeral fracture in older children and adolescents

The aim of the treatment of displaced supracondylar humeral fractures in children is to achieve a satisfactory reduction and firm fixation so that fractures can heal successfully, thus reducing the risk of iatrogenic injuries and complications. Closed reduction and percutaneous pinning is the standard treatment for supracondylar humeral fractures in children. Percutaneous pinning may be appropriate for younger children, but for older children (> 8 years old) and adolescents that have a larger body weight, there is an increased risk of secondary fracture displacement and elbow stiffness due to the limited fixed strength of the Kirschner wire and the need for supplementary plaster cast.

**Fig. 2:**
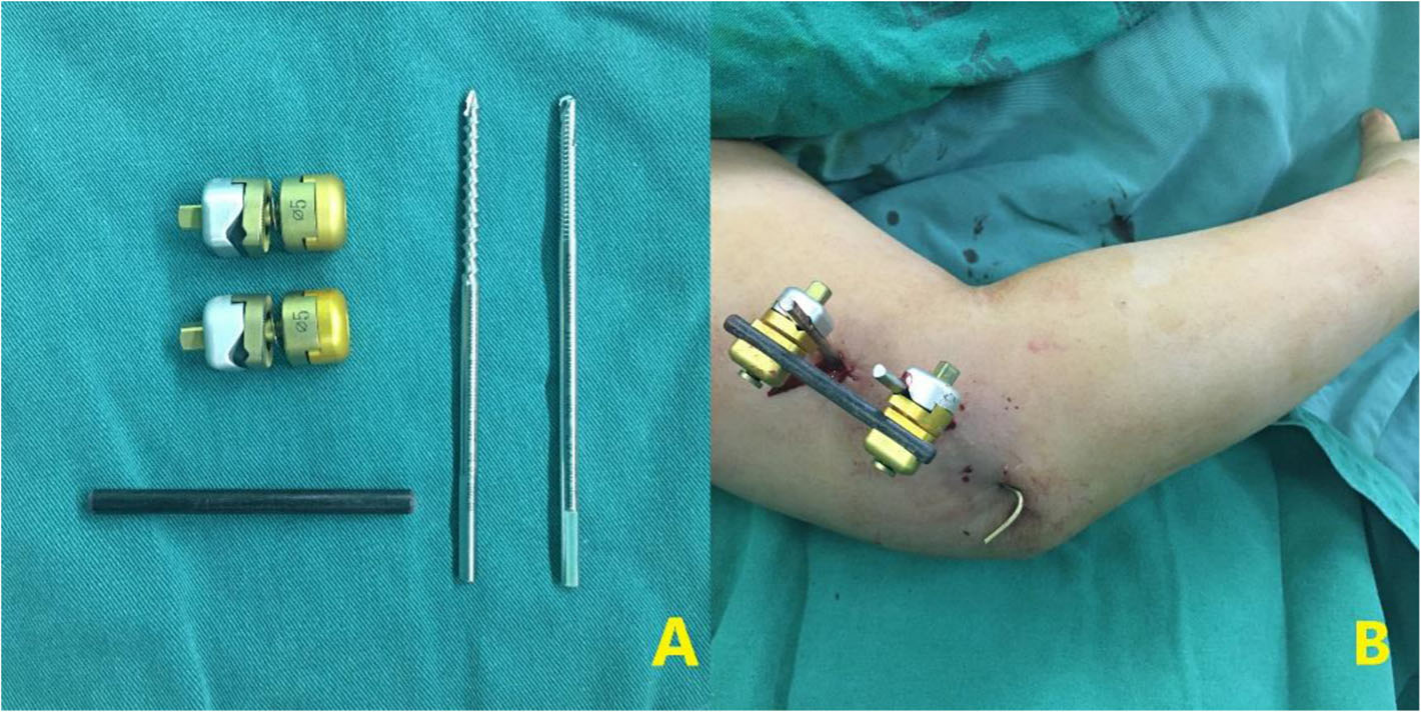
Composition and postoperative appearance of Slongo’s external fixation (**A**, **B**)

External fixation is a surgical alternative for supracondylar humeral fractures and was first proposed by Taller et al. [13]. Currently, there are three types of external fixation techniques used for supracondylar humeral fractures in children. The first type is an external fixation across the elbow to fix the proximal humerus fracture and the ulna, which was used by Gris [14] and Bogdan [15]. This form of fixation allows pronation and supination of the forearm; however, it does not allow flexion and extension of the elbow. The second type is the Ilizarov circular external fixation adopted by Gugenheim et al. [16]. In 2000, Gugenheim published satisfactory results in the treatment of supracondylar humeral fractures in children and adolescents. However, the operation technique of Ilizarov’s external fixation is complex, which limits its application. The third form is the unilateral external fixation form invented by professor Slongo without fixing the elbow joint [17]. Two Schanz screws are placed and connected at each end of the supracondylar humeral fracture and an anti-rotation Kirschner wire is inserted laterally. This surgical technique is relatively simple and a biomechanical study has shown that Slongo’s external fixation is more stable than the cross K-wire technique (Fig. 2) [18]. Indeed, the Schanz screw inserted can also assist in the reduction of fracture. Since 2016, Slongo’s external fixation has been applied in our treatment of supracondylar humeral fractures in older children and adolescents, showing good therapeutic efficacy. However, we also found that some children and adolescents may refuse to move the injured elbow due to postoperative pain and fear, which hindered the recovery of elbow function. Surgeons should identify strategies to overcome these fears and improve treatment for these patients. It is inadvisable to immobilize the elbow after Slongo’s external fixation since children with supracondylar humeral fractures can perform elbow activities after surgery that will improve their recovery.

### The significance of ultra-early rehabilitation in the treatment of supracondylar humeral fractures in older children and adolescents

Rehabilitation medicine greatly advanced in the rehabilitation of adult orthopedic surgery, but it is still limited in the field of children orthopedics. It is still debatable whether postoperative rehabilitation is necessary for children with supracondylar humeral fractures [19]. The supporting view is that children with supracondylar humeral fractures should receive rehabilitation treatment as soon as possible after the end of immobilization of the affected limb to reduce the risk of elbow stiffness [20]. Colović et al. [21] analyzed changes in elbow function after supracondylar humeral fractures and the effects of early rehabilitation in children by measuring elbows range of motion and using a modified DASH questionnaire. They found that early rehabilitation of supracondylar humeral fractures in children results in significantly better elbow function and that rehabilitation should start within 15 days after removal of the immobilization. In this regard, Keppler et al. [22] also found that rehabilitation helped children with supracondylar humeral fractures recover faster and that it did not increase the risk of complications. The risk of elbow stiffness after a supracondylar humeral fracture is significantly increased in older children and adolescents. Therefore, children older than 8 years and adolescents should be treated differently from younger children. We found that older children and adolescents subjected to ultra-early rehabilitation recovered faster, which allows patients to return to normal life. Moreover, early rehabilitation may help to alleviate psychological pressure on patients and their parents. Compared with the traditional fixation form of pinning and plaster cast, the use of an external fixation enables patients to perform early elbow function exercises. The results of this study showed that older children and adolescents with an external fixation had a satisfactory recovery of elbow function without elbow stiffness. Moreover, because a plaster cast was not required after the surgery, the affected elbow was able to move within the acceptable range of the children and their comfort was significantly increased. The clinical treatment effect of ultra-early rehabilitation was satisfactory in the follow-up. We also noticed that the difference in elbow ROM at 6 months between the rehabilitation group and control group was statistically significant. However, the ROM of the elbow in both groups had reached the ROM required for functional activity of daily life, the difference in ROM had no discernable impact on function. Regardless of postoperative rehabilitation, we conclude that there may be no significant difference in the final elbow function between the two groups in long-term follow-up.

A key limitation of this study is that this is a retrospective analysis. The incidence of supracondylar humeral fractures is relatively low in older children and adolescents. Therefore, we plan to further expand the number of patients and apply more appropriate rehabilitation techniques to the treatment of supracondylar humeral fractures in older children and adolescents. Future studies will focus on how to make the elbow function of children recover better and faster, which is in line with the concept of Enhanced Recovery After Surgery (ERAS) [23].

## Conclusions

Slongo’s external fixation is a safe and effective surgical treatment for supracondylar humeral fractures in older children and adolescents. It allows early postoperative elbow movement, avoids elbow stiffness, and ensures good fracture healing. The ultra-early rehabilitation treatment for older children and adolescents after Slongo’s external fixation can significantly speed up the recovery of the elbow joint, improve the quality of life of patients, and quickly enable them to return to normal life.

## Data Availability

All data generated or analyzed during this study are included in this manuscript.

## Abbreviations

ROM: Range of motion
CA: Carrying angle
